# Changes in bacterial community composition in the uterus of Holstein cow with endometritis before and after treatment with oxytetracycline

**DOI:** 10.1038/s41598-024-59674-4

**Published:** 2024-04-25

**Authors:** Xiao-Shi Cai, Hao Jiang, Jie Xiao, Xiangmin Yan, Penggui Xie, Wenjie Yu, Wen-fa Lv, Jun Wang, Xiangyu Meng, Cheng-zhen Chen, Mingjun Zhang, Yang Zhang, Bao Yuan, Jia-Bao Zhang

**Affiliations:** 1 College of Animal Sciences, Jilin University, Changchun, 130062 Jilin China; 2 College of Animal Husbandry Engineering, Henan Vocational College of Agriculture, Zhengzhou, 450002 Henan China; 3 Institute of Animal Husbandry, Xinjiang Academy of Animal Husbandry, Urumqi, 830001 Xinjiang China; 4 Yili Vocational and Technical College, Yili, 835000 Xinjiang China; 5 College of Animal Science and Technology, Jilin Agricultural University, Changchun, 130118 Jilin China; 6 Animal Husbandry Development Service Center of Tongyu County, Baicheng, 137200 Jilin China; 7 Changchun City, Jilin Province China

**Keywords:** Developmental biology, Microbiology, Diseases

## Abstract

It is important to study the bacteria that cause endometritis to identify effective therapeutic drugs for dairy cows. In this study, 20% oxytetracycline was used to treat Holstein cows (n = 6) with severe endometritis. Additional 10 Holstein cows (5 for healthy cows, 5 for cows with mild endometritis) were also selected. At the same time, changes in bacterial communities were monitored by high-throughput sequencing. The results show that *Escherichia coli, Staphylococcus aureus* and other common pathogenic bacteria could be detected by traditional methods in cows both with and without endometritis. However, 16S sequencing results show that changes in the abundance of these bacteria were not significant. Endometritis is often caused by mixed infections in the uterus. Oxytetracycline did not completely remove existing bacteria. However, oxytetracycline could effectively inhibit endometritis and had a significant inhibitory effect on the genera *Bacteroides*, *Trueperella*, *Peptoniphilus*, *Parvimonas*, *Porphyromonas*, and *Fusobacterium* but had no significant inhibitory effect on the bacterial genera *Marinospirillum*, *Erysipelothrix*, and *Enteractinococcus*. During oxytetracycline treatment, the cell motility, endocrine system, exogenous system, glycan biosynthesis and metabolism, lipid metabolism, metabolism of terpenoids, polyketides, cofactors and vitamins, signal transduction, and transport and catabolism pathways were affected.

## Introduction

Endometritis is a serious disease that affects reproductive performance, leads to low milk production and a low fertilization rate, increases risk of pregnancy loss, and severely reduces the production and economic benefits of dairy cow farming^1–3^. At calving the cervix is open, therefore environmental and reproductive tract microorganisms have the opportunity to ascend to and contaminate the uterus^4–6^. It is common for postpartum cows to have an inflammatory response in the uterus. Most cows can eliminate pathogen infections and resolve the inflammation through endometrial epithelial barrier and congenital immune defense functions within 4 weeks after delivery. However, this process will lead to pathological uterine inflammation due to poor management, physical damage, retention of fetal membranes, hormone disorders, denutrition, low immunity, and damage to the defense system^7–9^.

Studies have shown that pathogenic bacteria, including *Staphylococcus, Streptococcus, Escherichia, Corynebacterium, Pseudomonas, Proteus, Necrobacillus, Pseudomonas aeruginosa, Campylobacter genitalia, Haemophilus, Bacillus pyogenes, Bacterium burgeri, Trueperella, Fusobacterium, and Prevotella,* cause endometritis^10^. They are usually inhibited by antibiotics, hormones, and other drugs in clinical practice^11–13^.

Oxytetracycline (OTC) has good efficacy as an antibiotic for the treatment of endometritis^14–16^. OTC is an inexpensive, broad-spectrum antibiotic that is active against a wide variety of bacteria. OTC binds to the 30S ribosomal subunit and prevents the formation of an aminoacyl-tRNA-ribosome complex and further interferes with the ability of bacteria to produce proteins that are essential for bacteria to grow and multiply. However, some strains of bacteria have developed resistance to OTC, which has reduced its effectiveness for treating some types of infections^17–19^. Therefore, to effectively utilize the efficacy of OTC and treat endometritis, it is important to explore the bacterial communities that cause infection and are sensitive to OTC.

With the development of biotechnology, high-throughput sequencing has been widely used in clinical veterinary fields due to its high throughput, short sequencing time and low cost. When faced with certain clinical statuses, such as increased types of diseases, accelerated pathogen mutations, secondary infections, mixed infections, and latent infections, high-throughput sequencing has shown advantages in the areas of disease surveillance, rapid and accurate diagnosis, pathological research, and drug screening^20–22^.

This study used high-throughput sequencing to analyze changes in the bacterial community composition of bovine uterus contents in healthy Holstein cows, cows with endometritis, and cows with endometritis treated by OTC. Bacteria at the genus level that are easy or difficult to inhibit with OTC and the pathways that OTC affects were evaluated. These findings provide a theoretical basis for analyzing the pathogenesis of endometritis and the rational use of OTC.

## Results

### Pathogen identification in cultured bacteria

**Figure 1:**
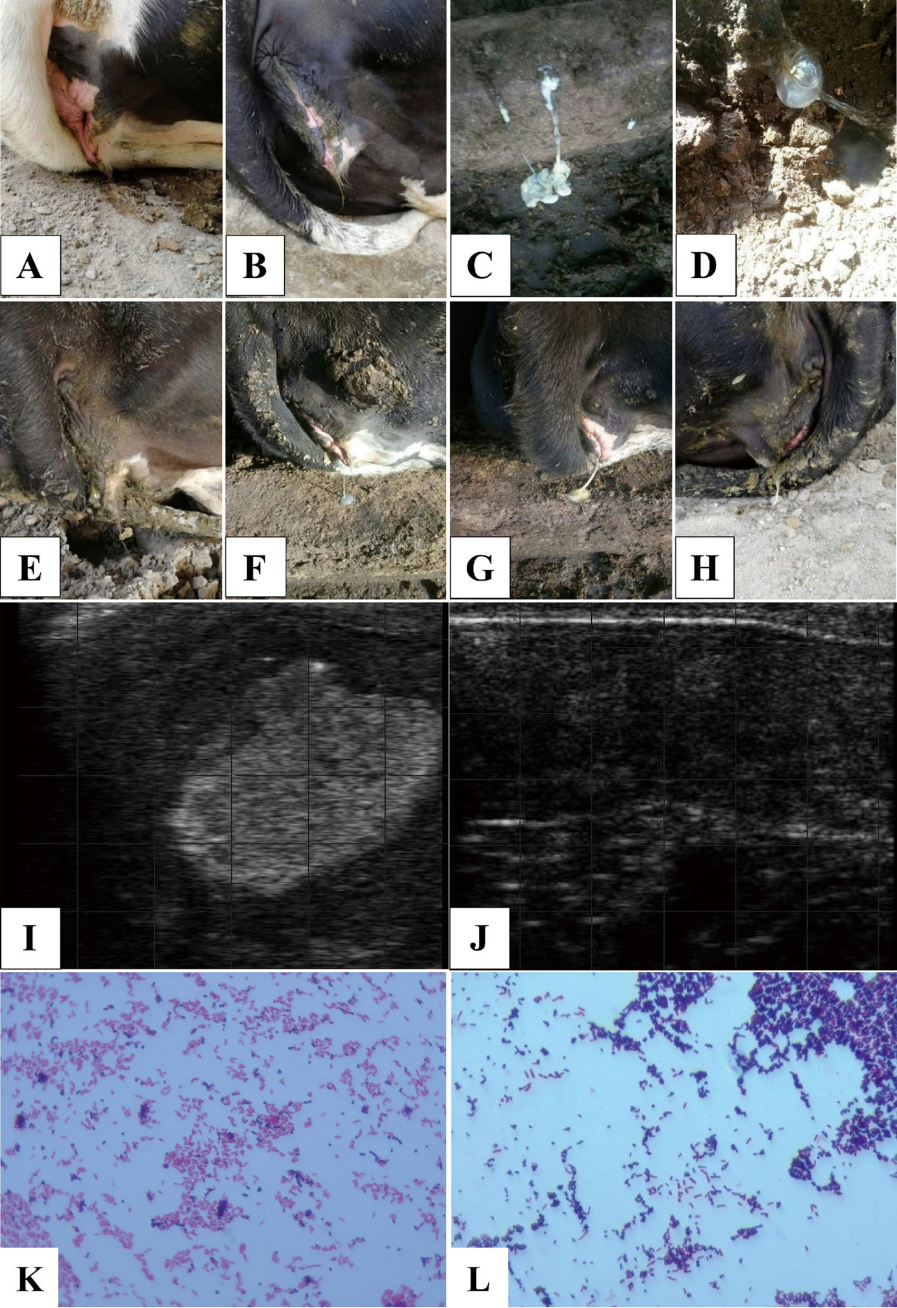
Observation, ultrasound, and microscopic imaging of endometritis and bacterial mixed infection of representative examples. Mildly diseased cows had red and swollen vulvae (**A**), and the vaginal discharge was slightly turbid and could not be drawn into filamentous mucus (**B**). A large amount of white (**C**) or yellow (**D**) purulent secretion with severe malodorous smell could be observed in the vaginal opening of severely diseased cows (**E**–**H**). The uterus of diseased cows showed abnormal ultrasound imaging with hyperechoic areas (**I**), while healthy or cured cows had clear black background (**J**). (**K**) Mixed infection with *Escherichia* and *Streptococcus* (magnification = 100×). (**L**) Mixed infection with *Escherichia* and *Staphylococcus* (magnification = 100×).

The detection rate of corresponding bacteria with common culture techniques in all four groups is shown in Table 1. Briefly, the bacteria detection rates (the number of positive samples to the number of all samples) between the NT and NC groups were similar. The detection rates of *Escherichia coli, Staphylococcus aureus, Staphylococcus haemolyticus*, and *Streptococcus uberis* were greater than 83%, while the detection rates of other bacteria were no less than 33%. The severity of endometritis can be quickly distinguished through visual observation and ultrasound methods (Fig. 1A–J). Microscopic examination results also showed that the cultures in this study had mixed infections of *Escherichia coli*, *Staphylococcus sp.,* and *Streptococcus sp.* (Fig. 1K, L). In individuals with severe endometritis (BT group), the detection rate of all mentioned bacteria in Table 1 is relatively higher. After treatment with oxytetracycline (AT group), the detection rate of mentioned bacteria was reduced. However, the related bacteria could still be detected in all samples.

**Table 1:** Pathogen identification in uterus.

Pathogens	Detection rates			
	NC^a^	NT^b^	BT^c^	AT^d^
*Escherichia coli*	3/5	3/5	6/6	2/6
*Staphylococcus aureus*	3/5	2/5	6/6	2/6
*Chryseobacterium indologenes*	2/5	2/5	2/6	1/6
*Moraxella osloensis*	2/5	3/5	3/6	1/6
*Bacillus cereus*	2/5	2/5	2/6	1/6
*Staphylococcus haemolyticus*	2/5	2/5	5/6	2/6
*Corynebacterium*	2/5	2/5	2/6	1/6
*Enterococcus faecium*	2/5	2/5	3/6	1/6
*Streptococcus uberis*	2/5	3/5	5/6	2/6

^a-d^NC = healthy cows (NC group), NT = mild diseased cows, BT = severely ill cows, AT = cows in BT group after OTC treatment.

### Alpha-diversity of bovine uterine bacterial communities

The sequencing reads of the bacterial 16S rRNA gene resulted in 1,488,649 clean tags from 22 samples. The evaluation results of each sample’s sequencing data are shown in Supplementary Table 1. These high-quality sequences clustered into an average of 1,117 bacterial OTUs. Numbers of identified phyla, classes, orders, families, genera, and species from these OTUs in all samples are shown in Supplementary Table 2.

**Figure 2:**
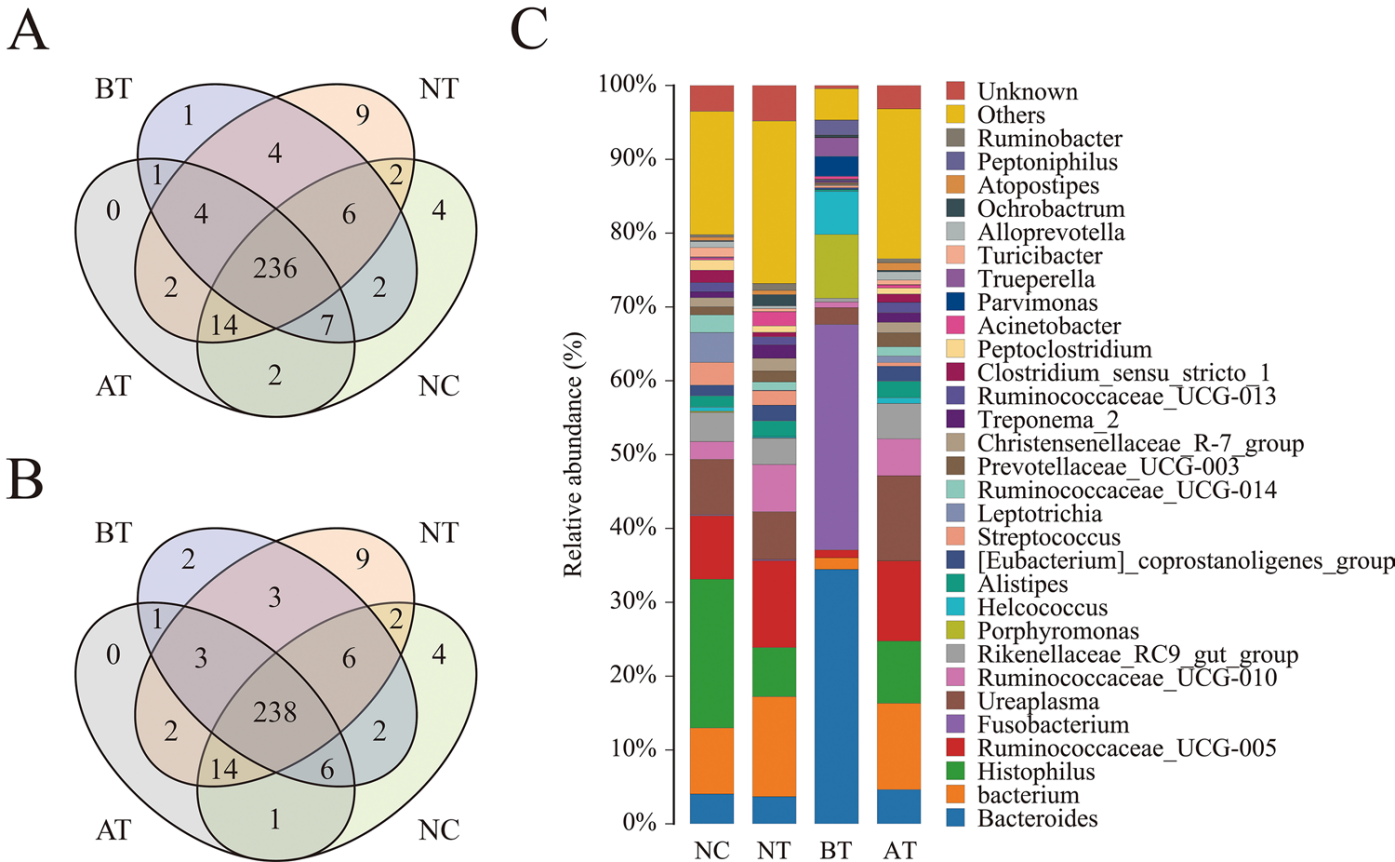
Venn diagram and abundances of the top 30 genera in the four groups. Venn diagrams showing the unique and shared genera of the bacterial communities across different groups at the genus (**A**) and species (**B**) levels. (**C**) Relative abundances of the top 30 bacterial genera in the four groups. Each color represents a genus, and the length of the patch represents the relative abundance ratio of all the bacterial communities in each sample. The other genera are merged in an “others” category. The category “unknown” represents a species that was not annotated with taxonomy.

**Figure 3:**
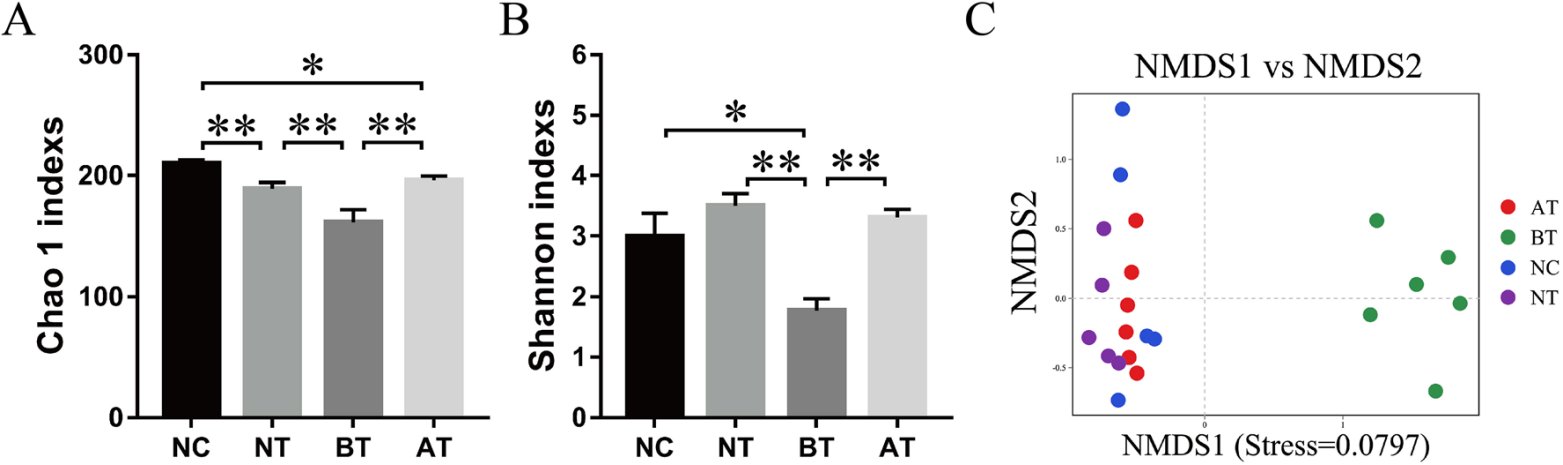
Variations in alpha-diversity and NMDS analysis among the four groups. (**A**) Comparisons of the bacterial community Chao1 indexes within the different groups. (**B**) Comparisons of the bacterial community Shannon indexes within the different groups. *, *P* < 0.05; **, *P* < 0.01 (with Student's t-test). (**C**) The dots in the figure represent the samples, and the different colors represent the different groups to which they belong. The distance between points indicates the degree of difference. Closer samples on the graph have greater similarity of diversity.

The number of unique/shared genera and species of the bacterial communities across different groups are shown in Fig. 2A,B. The genera *Histophilus* (20.16%), *Bacterium* (8.94%), *Ruminococcaceae_UCG-005* (8.61%), Ureaplasma (7.52%), and *Bacteroides* (4.04%) were the 5 most dominantly abundant bacteria in the NC group. *Bacterium* (13.56%), *Ruminococcaceae_UCG-005* (11.77%), *Histophilus* (6.65%), *Ureaplasma* (6.47%), and *Ruminococcaceae_UCG-010* (6.40%) were the 5 most dominantly abundant bacteria in the NT group. *Bacteroides* (34.46%), *Fusobacterium* (30.51%), *Porphyromonas* (8.65%), *Helcococcus* (5.85%), and *Parvimonas* (2.72%) were the 5 most dominantly abundant bacteria in the BT group. *Bacterium* (11.68%), *Ureaplasma* (11.50%), *Ruminococcaceae_UCG-005* (10.87%), *Histophilus* (8.43%), and *Ruminococcaceae_UCG-010* (5.00%) were the 5 most dominantly abundant bacteria in the AT group (Fig. 2C). The Chao1 and Shannon indexes were calculated for all samples. Alpha-diversity analysis showed significant differences among the four groups (Fig. 3A, B).

### Bacterial beta-diversity

**Figure 4:**
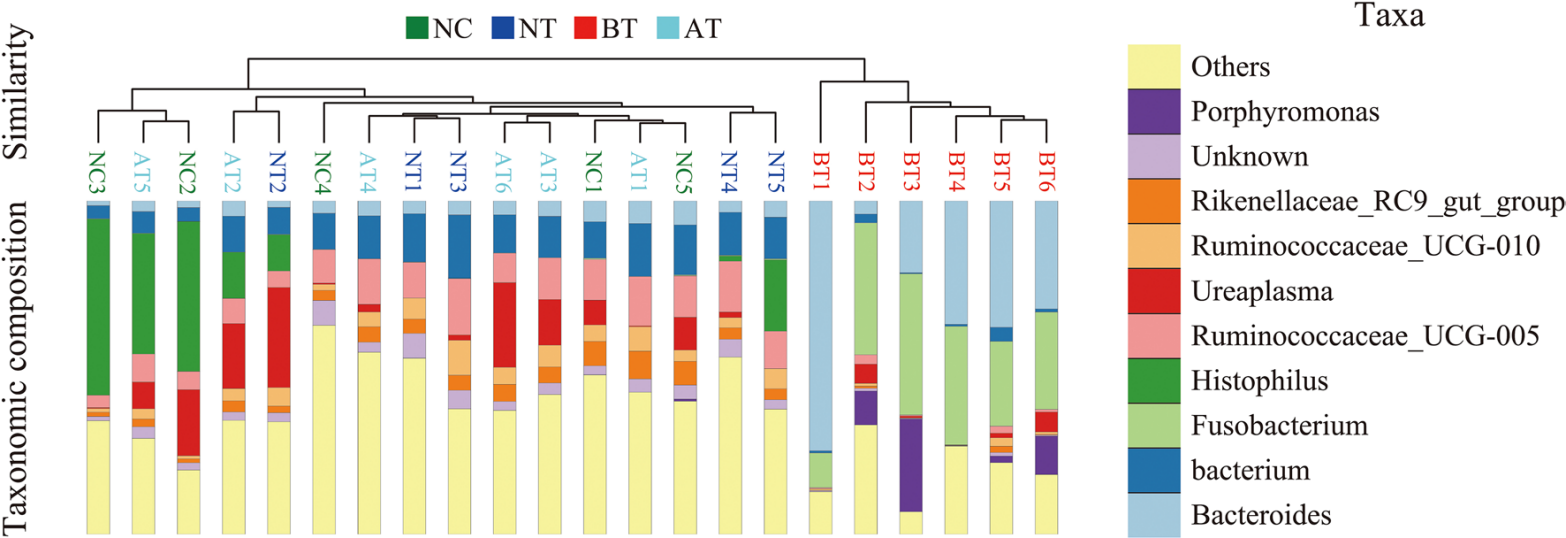
Bacterial beta diversity based on UPGMA clustering and abundances of the top 10 genera. To optimize the view, the histogram shows only the top 10 most abundant genera; the other genera are merged in an “others” category. “unknown” represents a species that are not annotated with taxonomy. Closer samples with shorter branch lengths have more similar genus-level compositions. Each color represents a genus, and the length of the patch represents the relative abundance ratio of all the bacterial communities in each sample.

A Bray–Curtis-based NMDS plot of all samples revealed a separation among the four groups (Fig. 3C). The Bray–Curtis-based distance and abundance metrics showed that samples belonging to the BT group had an obviously different distribution compared with that of the other three groups. A UPGMA-based hierarchical clustering analysis confirmed the alpha‐diversity analysis results, which showed that each group had unique communities compared with those of the other groups among the four groups (Fig. 4).

### Changes in the bacterial community in the uterus before and after treatment with OTC

**Figure 5:**
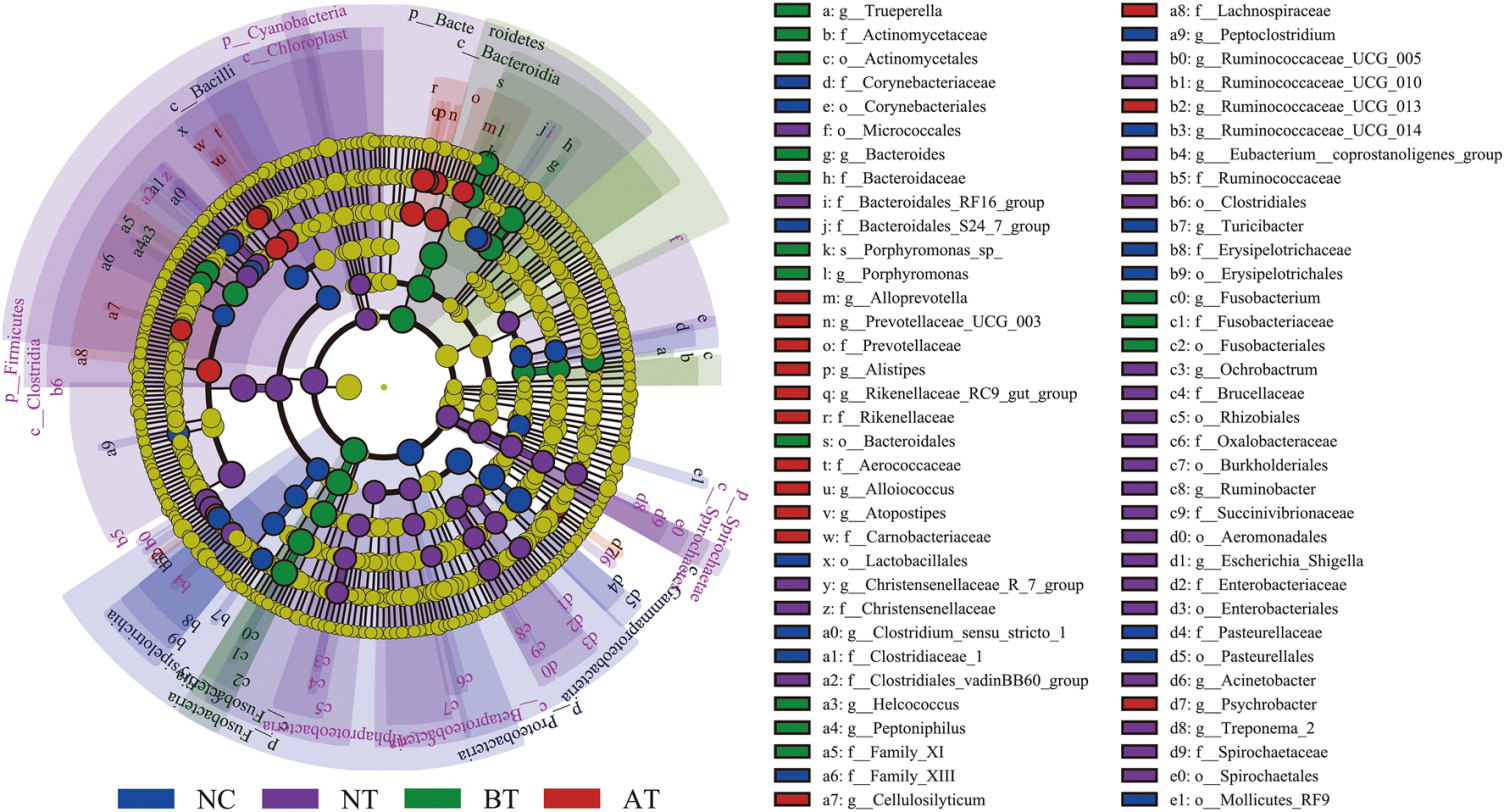
Significant changes of bacterial communities in the four groups as determined by LEfSe analysis. Taxonomic representation of statistically consistent differences within the four groups. The taxa with significantly different abundances among groups are represented by colored dots. Colored areas (NC, NT, BT, and AT) mark the most prominent bacteria found in this study. The cladogram was generated by LEfSe and indicates differences at the phylum, class, family, order, genus, and species levels in the four groups. Each successive circle represents a phylogenetic level. Only taxa meeting a linear discriminant analysis (LDA) significance threshold of > 3.5 are shown.

The results of LEfSe analysis indicated that the bacterial communities with significant abundance changes in the four groups included 6 phyla, 10 classes, 15 orders, 24 families, 28 genera, and 1 species (Fig. 5 and Supplementary Table 3). Metagenomic analysis showed that the bacterial composition varied in uteruses with endometritis after OTC treatment.

**Figure 6:**
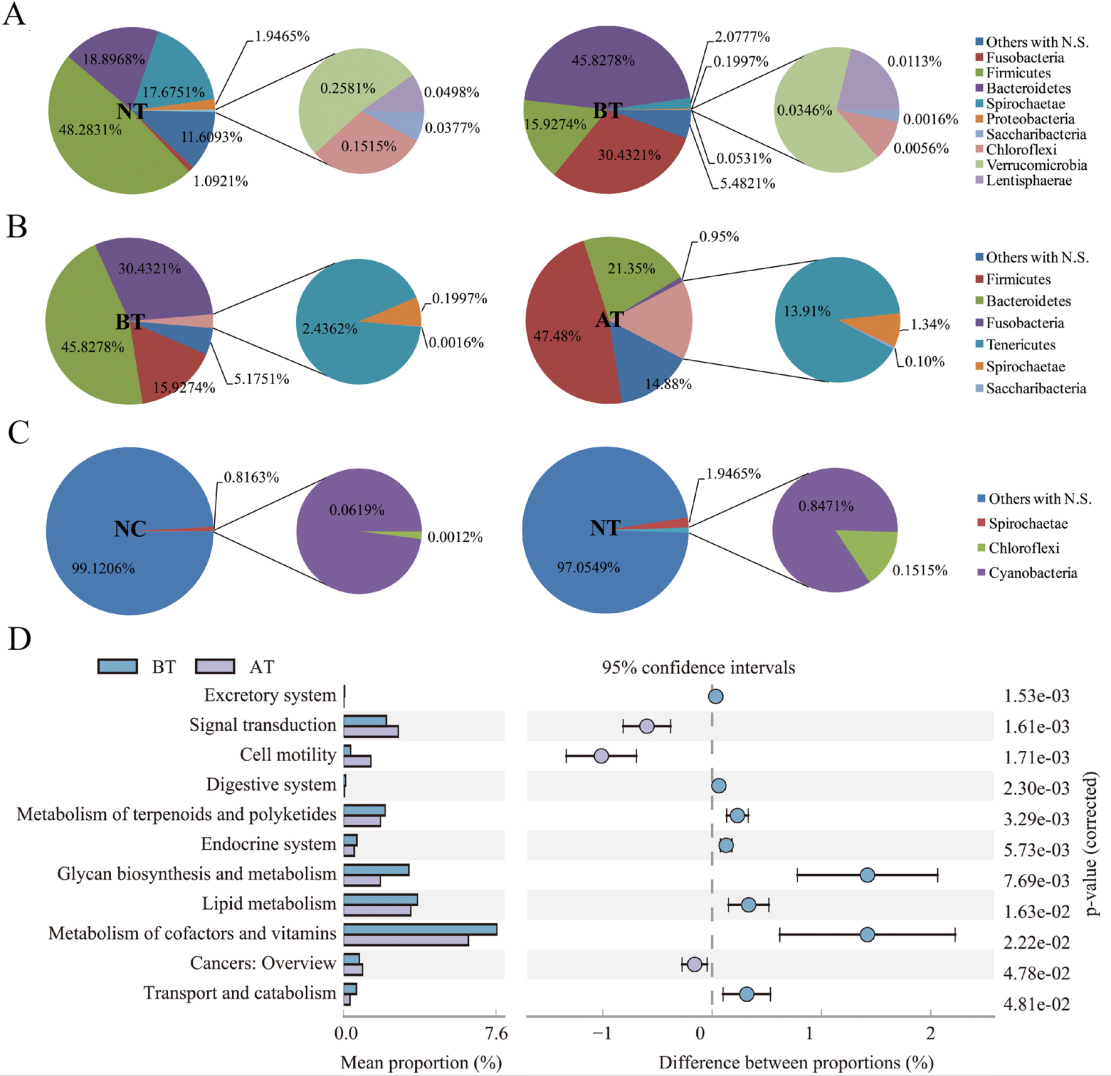
Abundances of bacterial communities changes at phylum level and related metabolic pathways with OTC treatment. (**A**–**C**) Relative abundances of bacterial phyla in four groups. Each color represents a phylum. No significantly changed phyla are merged in an “Others with N.S.” category. (**D**) KEGG pathways associated with significantly different abundances in the bacterial metagenomics profiles of BT and AT groups. The bars represent the proportion of each category in the data. Category differences with a *P* < 0.05 were considered to be significant.

We first clarified the changes in the bacterial communities at the phylum level (Fig. 6A–C). The relative abundance of *Fusobacteria*, *Firmicutes*, *Bacteroidetes*, *Spirochaetae*, *Proteobacteria*, *Saccharibacteria*, *Chloroflexi*, *Verrucomicrobia*, and *Lentisphaerae* varied significantly between the NT and BT groups (*P* < 0.05). The relative abundance of *Fusobacteria*, *Firmicutes*, *Spirochaetae*, *Saccharibacteria*, *Bacteroidetes*, and *Tenericutes* varied significantly between the BT and AT groups (*P* < 0.05). The relative abundance of *Chloroflexi*, *Cyanobacteria*, and *Spirochaetae* varied significantly between the NT and NC groups (*P* < 0.05). No significant bacterial community abundance changes occurred between the NC and AT groups at the phylum level.

The top 5 increased/decreased and unique genera in the four groups are shown in Table 2, Supplementary Table 4, and Supplementary Table 5. The relative abundances of *Clostridium_sensu_stricto_6* and *Tissierella* were much higher (25.839 and 10.665 times, respectively) in the NC group than in the NT group, while those of *Tepidimonas*, *Ornithinicoccus*, *Paludibacter*, *Serratia*, and *Rhizobium* were much lower (0.016, 0.018, 0.023, 0.042, and 0.043 times, respectively). *Sulfuricurvum*, *Caproiciproducens*, and *Lactobacillus* could not be detected in the NC group, while *Paraliobacillus* and *Amphibacillus* were undetectable in the NT group. The abundances of *Marinospirillum*, *Erysipelothrix*, *Enteractinococcus*, *Erysipelotrichaceae_UCG-009*, and *Papillibacter* were much lower in the BT group than in the AT group (0.002, 0.005, 0.008, 0.010, and 0.012 times, respectively), while those of *Fusobacterium*, *Porphyromonas*, *Parvimonas*, *Peptoniphilus*, and *Trueperella* were much higher (1,548.265, 1,022.113, 415.864, 233.766, and 92.739 times, respectively). *Faecalibacterium*, *hoa5-07d05_gut_group*, *Salinicoccus*, *Methylophilus*, *Proteiniclasticum*, *Cellulomonas*, *Amphibacillus*, and *Phenylobacterium* were undetectable in the BT group.

**Table 2:** Variations of dominant bacteria composition.

NC versus NT		BT versus AT	
Bacteria	Abundance change (times)	Bacteria	Abundance change (times)
Tepidimonas	0.016	Marinospirillum	0.002
Ornithinicoccus	0.018	Erysipelothrix	0.005
Paludibacter	0.023	Enteractinococcus	0.008
Serratia	0.042	Erysipelotrichaceae_UCG-009	0.010
Rhizobium	0.043	Papillibacter	0.012
Tissierella	10.665	Trueperella	92.738
Clostridium_sensu_stricto_6	25.839	Peptoniphilus	233.766
Sulfuricurvum	+ /−	Parvimonas	415.864
Caproiciproducens	+ /−	Porphyromonas	1022.113
Lactobacillus	+ /−	Fusobacterium	1548.265
Paraliobacillus	−/ +	Faecalibacterium	−/ +
Amphibacillus	−/ +	Hoa5-07d05_gut_group	−/ +
		Salinicoccus	−/ +
		Methylophilus	−/ +
		Proteiniclasticum	−/ +
		Cellulomonas	−/ +
		Amphibacillus	−/ +
		Phenylobacterium	−/ +

" + " represents bacteria can be detected while "−" represents bacteria is undetectable.

BT = severely ill cows, AT = cows in BT group after OTC treatment.

### Changes in functional pathways of bacterial communities among the different groups

As shown in Fig. 6D, there were 11 biological pathways (including excretory system, signal transduction, cell motility, digestive system, metabolism of terpenoids and polyketides, endocrine system, glycan biosynthesis and metabolism, lipid metabolism, metabolism of cofactors and vitamins, cancers: overview, and transport and catabolism) involved in bacterial communities with significant abundance changes between the BT and AT groups.

## Discussion

OTC has a good therapeutic effect on endometritis, but some bacteria are resistant to OTC. However, most research on the use of oxytetracycline (OTC) in the treatment of endometritis focus on specific flora, especially the culturable bacteria^23,24^. The inhibition effects of OTC on the metagenomic-based analysis, especially non-culturable bacteria, and its effects on the biological functions of the corresponding bacteria are not well known.

In this study, we isolated and identified several common bacteria that cause endometritis. Overall, our analysis showed that the microbial communities in cows with endometritis are similarly dynamic within either animal groups (Figs. 3, 4, Supplementary Table 4 and 5) as reported by other research^25^. It is noteworthy that although OTC stimulated the elimination of Bacteroidetes, Fusobacteria, etc., it did not eliminate all bacterial species. On the contrary, some bacteria species increased after OTC administration. The detection rates of bacteria such as *Chryseobacterium indologenes*, *Bacillus cereus*, and *Corynebacterium* were even higher in the NC group than in the BT group. Although some bacteria (e.g. Escherichia coli, Staphylococcus aureus, Staphylococcus haemolyticus, and Streptococcus), which was considered as pathogenic bacteria, has high detection rate or proportion, the individuals have not shown disease characteristics or have been cured. This indicates that identification by traditional bacterial culture methods has limitations for endometritis infection-associated strain identification. In addition, simply analyzing the etiology or treatment of endometritis based on a specific pathogenic bacterial population may also be inaccurate.

At the phylum level, changes in the bacterial communities between the NT group and the BT group indicated that abundances of *Fusobacteria*, *Firmicutes*, *Bacteroidetes*, *Spirochaetae*, *Proteobacteria*, *Saccharibacteria*, *Chloroflexi*, *Verrucomicrobia*, and *Lentisphaerae* changed the most from mild symptoms to severe illness. The changes in bacterial communities between the BT and AT groups indicated that OTC could significantly inhibit *Bacteroidetes* and *Fusobacteria*. The changes in bacterial communities between the NC and NT groups indicated that endometritis begins with changes in the abundance of *Spirochaetae*, *Chloroflexi*, and *Cyanobacteria*. These results seem to be contradictory. For example, *Streptococcus* and *Staphylococcus* belong to the *Firmicutes* phylum; *Escherichia coli* belongs to the *Proteobacteria* phylum. The abundance of Firmicutes was increased, while *Proteobacteria* did not change significantly after OTC treatment. This result suggests that exploring the bacteria that might lead to endometritis at the phylum level as a guideline to prevent or cure endometritis is ambiguous. However, since the database is not sufficiently comprehensive, we selected the genus level to further annotate the differences between different groups of bacteria and analyze their biological effects.

At the genus level, *Sulfuricurvum*, *Caproiciproducens*, and *Lactobacillus* were not detected in the uterus of healthy cattle but were in cows in the onset stage of endometritis. The abundances of bacteria such as *Tepidimonas*, *Ornithinicoccus*, and *Paludibacter* increased significantly during the deterioration of endometritis. Interestingly, *Amphibacillus* and *Paraliobacillus* could not be detected in the uteruses with endometritis. *Amphibacillus* was detectable in cows with endometritis until they were cured. *Faecalibacterium*, *hoa5-07d05_gut_group*, *Salinicoccus*, *Methylophilus*, *Proteiniclasticum*, *Cellulomonas*, and *Phenylobacterium* were undetectable when cows were severely ill, indicating that these bacteria could not survive in a severe endometritis environment.

In addition, we also found that no flora disappeared after OTC treatment, indicating that OTC could not completely remove existing bacteria. However, after treatment with OTC, the relative abundances of *Bacteroides*, *Trueperella*, *Peptoniphilus*, *Parvimonas*, *Porphyromonas*, and *Fusobacterium* decreased to 15.5824%, 1.0783%, 0.4276%, 0.0979%, and 0.0646%, respectively, indicating that OTC has an active inhibitory effect against these bacteria. In contrast, the relative abundance of *Marinospirillum*, *Erysipelothrix*, and *Enteractinococcus* increased tens of thousands of times after OTC treatment, indicating that OTC had no significant inhibitory effect on these bacteria.

Finally, we analyzed the possible biological pathways that OTC affected and its potential effects on the physiological role of uterine bacterial communities. Postpartum insufficient uterine involution, inflammatory secretions in the uterine cavity and poor drainage are important hidden dangers of endometritis. Bacteria lead to endometritis through biological pathways such as the excretory system, endocrine system, signal transduction, and cell motility^26–28^. The use of OTC can affect the utilization of glycan and lipids in bacterial communities and affect the content of metabolites (including terpenoids and polyketides), thereby affecting the adaptability and reproductive capacity of the communities^29–31^. In addition, OTC also affects the ability of bacteria to adapt to diverse conditions, which is mediated by regulation of encoded signal transducers (or their fraction in the total protein set)^32^. These results explain why OTC effectively reduced the production of cytokines, which actively participate in the inflammatory process in the uterus^27,33^, and had a potential role in responses initiated by recognition proteins, which, in the presence of foreign organisms including bacteria, trigger distinct signal transduction and modulation pathways^34^. More importantly, we also found that the treatment with OTC had a significant impact on the metabolism of cofactors and vitamins pathway. This result can also explain why the long-term use of OTC causes changes in intestinal communities and causes a vitamin imbalance. Therefore, vitamin supplementation is often used when treating animals with OTC^35,36^.

In conclusion, this study identified changes in bacteria at the genus level in uteri of cows with endometritis before and after OTC treatment. During OTC treatment, the excretory system, signal transduction, cell motility, digestive system, metabolism of terpenoids and polyketides, endocrine system, glycan biosynthesis and metabolism, lipid metabolism, metabolism of cofactors and vitamins, and transport and catabolism pathways were involved. OTC had a significant inhibitory effect on the genera *Bacteroides*, *Trueperella*, *Peptoniphilus*, *Parvimonas*, *Porphyromonas*, and *Fusobacterium* but had no significant inhibitory effect on bacteria such as *Marinospirillum*, *Erysipelothrix*, and *Enteractinococcus*.

## Methods

All methods (including all animal experiments) in this study were performed in accordance with the relevant guidelines and approved by the Institutional Animal Care and Use Committee of Jilin University (IACUC-ID-SY201801026). This study is reported in accordance with ARRIVE guidelines.

## Experimental animals

On a dairy farm with 442 cows, a total of 16 Holstein cows with same months of age and parity order were detected. According to the degree of illness, among all the samples, 5 cows were healthy cows (NC group), and 5 cows had mild disease (NT group). Six cows were severely ill (BT group) and became healthy after oxytetracycline hydrochloride (OTC; Aladdin, Shanghai, China) treatment (AT group).

### Uterine contents collection

Before sample collection, the anus and vulva area were wiped and disinfected with a 5% potassium permanganate solution, and then the vulvae were disinfected with cotton swabs moistened with 70% alcohol. Endometrial swabs were used to collect the uterine contents as described with some modification^25,37^. In general, an improved ethylene oxide-sterilized human uterine sampling brush was placed into an infusion cannula, and advanced into the uterus and gently rotated^38^. After removal, the uterine sampling swabs was rinsed with sterile saline, and the solution samples were collected in two sterile tubes. Bacteria in one tube were stored immediately at − 80 °C until DNA extraction. Bacteria in another tube were prepared for pathogen identification.

### Diagnosis of endometritis

Holstein cows with endometritis were identified based the visual observation and ultrasound testing as described with some modification. In general, cows with a foul-smelling and slightly turbid discharged vaginal mucus were diagnosed with mild disease. Cows with fetid and white/yellow vaginal purulent secretions were diagnosed as seriously diseased. Through rectal palpation, the volume of seriously diseased cow's uterine horn was increased and often accompanied by a certain degree of edema, the elasticity of the cervix and uterus deteriorated. Through ultrasound examination, cows with thickened uterine wall and irregular inner edge as well as an obvious fluid sonolucent area in the uterus were identified as diseased cow while healthy or cured cows had clear black background without significant hyperechoic areas (Fig. 1).

### Isolation and biochemical identification of endometritis pathogens

Broth medium, MacConkey agar medium, blood agar base medium, and related media were obtained from Qingdao Hope Bio-Technology (Qingdao, China). Bacteria in uterine contents were cultured with the corresponding media at 37 °C incubator for 24 h for Gram staining and microscopic examination. The morphology, staining characteristics and growth characteristics of the colonies were observed and classified. Each sample was subjected to 5 biological replicate tests, and any positive results indicated that the corresponding bacteria existed in the uterine contents. The isolated bacteria obtained were identified based on morphology, Gram staining reactions and different biochemical tests using Bergey’s manual for determinative bacteriology^39^.

### OTC treatment and efficacy evaluation

Briefly, uterine perfusion was performed on a diseased cow with a 20% concentration of OTC (200 g of OTC in 1 L of sterile saline). The vulva was cleaned and disinfected, and then 20 mL of OTC was pushed into the uterus every 2 days by a syringe through an infusion cannula. Four episodes of perfusion was considered a treatment course. One week after stopping the treatment, a rectal examination and an ultrasound examination were performed to verify the curative effect. When a white/yellow stench of vaginal purulent secretions was still found from the *orificium vaginae* and an inflammatory area was detected by ultrasound examination, the treatment was ineffectual (Fig. 1I). When there was no congestion in the vaginal mucosa, the uterine mucus was clear, and there were abnormal findings on ultrasound, the cows were considered cured (Fig. 1J).

### DNA extraction and sequencing

Total bacterial and fungal DNA were extracted from samples using a PowerSoil DNA Isolation Kit (MoBio Laboratories, CA, USA) according to the manufacturer's protocol. Then, DNA was stored at − 80 °C until further processing.

Common primer pairs combined with adapter sequences and barcode sequences were used to amplify the bacterial 16S rRNA gene (forward primer, 5'-ACTCCTACGGGAGGCAGCA-3'; reverse primer, 5'-GGACTACHVGGGTWTCTAAT-3'). PCR amplification was performed in a total volume of 50 μL, which contained 0.2 μL of Q5 High-Fidelity DNA Polymerase (New England Biolabs Inc., MA, USA), 10 μL of buffer, 10 μL of High GC Enhancer, 1 μL of dNTPs, 10 μM each primer and 60 ng of genomic DNA. Thermal cycling conditions were as follows: an initial denaturation at 95 °C for 5 min, followed by 15 cycles at 95 °C for 1 min, 50 °C for 1 min and 72 °C for 1 min, with a final extension at 72 °C for 10 min. PCR products from the first step PCR were purified through VAHTSTM DNA Clean Beads (Vazyme, Nanjing, China). A second round of PCR was then performed in a 40 μL reaction that contained 20 μL of 2 × Phusion HF Mix (Thermo Fisher Scientific, CA, USA), 8 μL of ddH_2_O, 10 μM each primer and 10 μL of PCR products from the first step. Thermal cycling conditions were as follows: an initial denaturation at 98 °C for 30 s, followed by 10 cycles at 98 °C for 10 s, 65 °C for 30 s min and 72 °C for 30 s, with a final extension at 72 °C for 5 min. Finally, all PCR products were quantified by a Quant-iT™ dsDNA HS Assay Kit (Thermo Fisher Scientific) and pooled together.

High-throughput sequencing analysis of bacterial and fungal genes was performed on the purified, pooled samples using the Illumina HiSeq 2500 platform (2 × 250 paired-end sequencing; Biomarker Technologies Corporation, Beijing, China).

### Bioinformatics analyses

Raw sequences were generated from the Illumina HiSeq sequencing platform.Quality control and taxon classification were performed with FLASH v1.2.7 (http://ccb.jhu.edu/software/FLASH/), Trimmomatic (http://www.usadellab.org/cms/?page=trimmomatic), and UCHIME v4.2 (http://drive5.com/usearch/manual/uchime_algo.html). The sequences were further analyzed with the QIIME software package (http://qiime.org/). Operational taxonomic units (OTUs) were selected using a de novo OTU-picking protocol with a 97% identity threshold, and then, a representative sequence was picked for each OTU using Mothur software (https://www.mothur.org/) and the Silva database (https://www.arb-silva.de/) to annotate taxonomic information for each representative sequence.

Alpha-diversity, including Chao1 and Shannon indexes, was first calculated by Mothur software. Then, we compared overall samples between intragroup compositions using the unweighted pair-group method with arithmetic mean (UPGMA, http://genomes.urv.cat/UPGMA/), Venn^40^, and nonmetric multidimensional scaling (NMDS)^41^. Species with significant differences in microbial community composition abundance between groups were then analyzed with line discriminant analysis effect size (LEfSe, http://huttenhower.sph.harvard.edu/galaxy)^42^ and Metastats (http://metastats.cbcb.umd.edu/)^43^ methods. PICRUSt (http://picrust.github.com/picrust/)^44^ and Kyoto Encyclopedia of Genes and Genomes (KEGG, https://www.kegg.jp/)^45–47^ were used to predict function changes caused by bacterial microbial communities between the BT and AT groups based on the R language platform (R Core Team, https://www.r-project.org/).

### Supplementary Information

Supplementary Information.

## Data Availability

The data presented in this study are available in the article or Supplementary Material. The raw sequencing data generated and analysed during the current study are available in the Sequence Read Archive (SRA) repository (SAMN35443045–SAMN35443066) under BioProject PRJNA976827.
