# Supplementary material for: Changes in bacterial community composition in the uterus of Holstein cow with endometritis before and after treatment with oxytetracycline
Source: Sci Rep. 2024 Apr 25;14:9511. doi: 10.1038/s41598-024-59674-4 (PMC11045718; doi:10.1038/s41598-024-59674-4)
Supplement: Supplementary file 1 — Supplementary Information. [file 41598_2024_59674_MOESM1_ESM.zip › Supplementary Table 1. Sequencing quality of all samples.docx]

Supplementary Table 1. Sequencing quality of all samples

| Sample ID | PE  Reads | Raw  Tags | Clean  Tags | Effective  Tags | AvgLen  (bp) | Q30  (%) | Effective  (%) |
| --- | --- | --- | --- | --- | --- | --- | --- |
| NC1 | 80236 | 76396 | 68615 | 66323 | 417 | 95.08 | 82.66 |
| NC2 | 79956 | 76001 | 67854 | 65193 | 424 | 94.76 | 81.54 |
| NC3 | 80027 | 76693 | 68948 | 68344 | 421 | 94.66 | 85.4 |
| NC4 | 80097 | 75753 | 67710 | 66717 | 415 | 94.99 | 83.3 |
| NC5 | 80197 | 76261 | 68542 | 67078 | 416 | 94.99 | 83.64 |
| NT1 | 79803 | 75728 | 68314 | 62822 | 415 | 94.86 | 78.72 |
| NT3 | 79775 | 75835 | 67860 | 57523 | 418 | 94.68 | 72.11 |
| NT2 | 79821 | 74948 | 66630 | 55266 | 422 | 94.66 | 69.24 |
| NT4 | 79866 | 76496 | 69144 | 66499 | 415 | 95.01 | 83.26 |
| NT5 | 79934 | 75220 | 66702 | 53325 | 415 | 94.69 | 66.71 |
| BT1 | 80122 | 75320 | 67105 | 65001 | 421 | 95.08 | 81.13 |
| BT2 | 79740 | 75014 | 67487 | 66494 | 412 | 94.99 | 83.39 |
| BT3 | 79998 | 73972 | 66046 | 64699 | 416 | 94.86 | 80.88 |
| BT4 | 79930 | 74899 | 67261 | 66530 | 414 | 94.93 | 83.24 |
| BT5 | 79645 | 74540 | 66407 | 63739 | 416 | 94.91 | 80.03 |
| BT6 | 80221 | 75134 | 67057 | 63755 | 417 | 94.99 | 79.47 |
| AT1 | 80145 | 75698 | 67956 | 66535 | 414 | 94.93 | 83.02 |
| AT2 | 80009 | 75996 | 68120 | 66452 | 417 | 94.93 | 83.06 |
| AT3 | 79988 | 75905 | 68108 | 65642 | 417 | 94.91 | 82.06 |
| AT4 | 80025 | 75862 | 67929 | 64507 | 421 | 94.6 | 80.61 |
| AT5 | 80095 | 76201 | 68250 | 67363 | 419 | 95.01 | 84.1 |
| AT6 | 80035 | 74938 | 66604 | 62758 | 419 | 94.81 | 78.41 |
