# Supplementary material for: Changes in bacterial community composition in the uterus of Holstein cow with endometritis before and after treatment with oxytetracycline
Source: Sci Rep. 2024 Apr 25;14:9511. doi: 10.1038/s41598-024-59674-4 (PMC11045718; doi:10.1038/s41598-024-59674-4)
Supplement: Supplementary file 1 — Supplementary Information. [file 41598_2024_59674_MOESM1_ESM.zip › Supplementary Table 2. Bacterial OTUs in each taxonomic grade.docx]

Supplementary Table 2. Bacterial OTUs in each taxonomic grade

| Sample | Phylum | Class | Order | Family | Genus | Species |
| --- | --- | --- | --- | --- | --- | --- |
| NC1 | 1 | 11 | 25 | 50 | 82 | 198 |
| NC2 | 1 | 13 | 24 | 50 | 88 | 201 |
| NC3 | 1 | 14 | 29 | 53 | 88 | 188 |
| NC4 | 1 | 15 | 28 | 50 | 87 | 197 |
| NC5 | 1 | 13 | 24 | 49 | 83 | 199 |
| NT1 | 1 | 15 | 28 | 51 | 87 | 200 |
| NT2 | 1 | 14 | 24 | 47 | 81 | 171 |
| NT3 | 1 | 13 | 25 | 47 | 84 | 184 |
| NT4 | 1 | 17 | 31 | 54 | 92 | 196 |
| NT5 | 1 | 14 | 27 | 47 | 83 | 181 |
| BT1 | 1 | 13 | 23 | 44 | 77 | 144 |
| BT2 | 1 | 14 | 26 | 47 | 78 | 166 |
| BT3 | 1 | 13 | 26 | 41 | 72 | 129 |
| BT4 | 1 | 12 | 24 | 40 | 70 | 119 |
| BT5 | 1 | 14 | 29 | 53 | 89 | 196 |
| BT6 | 1 | 13 | 23 | 44 | 76 | 139 |
| AT1 | 1 | 13 | 25 | 47 | 85 | 193 |
| AT2 | 1 | 13 | 23 | 46 | 81 | 180 |
| AT3 | 1 | 13 | 24 | 50 | 88 | 203 |
| AT4 | 1 | 12 | 24 | 49 | 83 | 185 |
| AT5 | 1 | 13 | 27 | 50 | 84 | 187 |
| AT6 | 1 | 12 | 24 | 51 | 90 | 202 |
| Total | 1 | 18 | 36 | 63 | 109 | 253 |
